# 

**Published:** 1816-04

**Authors:** 


### MONTHLY CATALOGUE OF MEDICAL BOOKS.

OBSERVATIONS, with Cases, illustrative of a new, simple, and expeditious Mode of Curing Rheumatisms and Sprains. By William Balfour, M.D.—Underwood.

An Experimental Inquiry into the Nature, Cause, and Varieties of the Arterial Pulse. By C. H. Parry, M.D. F.R.S. 8vo.—Underwood.

A Narrative of a Journey to London in 1814, or a Parallel of the English and French Surgery; preceded by some Observations on the London Hospitals. By P. J. Roux. 8vo.—Cox and Son.

Rudiments of the Anatomy and Physiology of the Human Body. By T. J. Armiger. 8vo.—Cox and Son.

Health, a Poem; shewing how to Procure, Preserve, and Restore it. To which is annexed, the Doctor's Decade. By E. Baynard, M.D. 12mo.

A Practical Treatise on the Diseases of the Foot of the Horse. By R. H. Budd, Veterinary Surgeon. 8vo.—Longman and Co.

The Anatomy and Physiology of the Human Body. By John and Charles Bell. The fourth edition, in 3 vols. 8vo.—Longman and Co.

An Essay on the Bots of Horses and other Animals. By Bracy Clarke, F.L.S. Veterinary Surgeon. 4to.—Callow.

Instructions to Parents on the Management of their Children. By John Way, Surgeon. 12mo.—Callow.

---

### *NOTICES TO CORRESPONDENTS.*

*We have been favoured with Communications from* Drs. PEARSON, EDGELL, FISKE; R. N. STARR, Esq.; An OLD CORRESPONDENT; *&c. &c.*

*Our Correspondents will please to be particular in the Address of their Communications, which should be thus,* "To the EDITORS of the LONDON MEDICAL and PHYSICAL JOURNAL, at Mr. SOUTER'S, No. 1, Paternoster-Row; or at Mr. ADLARD'S, 23, Bartholomew-Close."

